# Forgotten but Not Gone! Syphilis Induced Tenosynovitis

**DOI:** 10.1155/2016/7420938

**Published:** 2016-12-06

**Authors:** Felicia Ratnaraj, David Brooks, Mollie Walton, Arun Nagabandi, Mahmoud Abu Hazeem

**Affiliations:** CHI Health Creighton University Medical Center, Omaha, NE, USA

## Abstract

*Objective*. Tenosynovitis, inflammation of a tendon and its synovial sheath, is a rare manifestation of secondary syphilis and if diagnosed early is reversible.* Background*. A 52-year-old male with past medical history of untreated syphilis presented with gradual onset of swelling and pain of the right fourth metacarpophalangeal joint (MCP). He reported a history of painless penile lesions after having sexual intercourse with a new partner approximately five months ago which was treated with sulfamethoxazole/trimethoprim. An RPR done at that time came back positive with a high titer; however, patient was lost to follow-up. On examination, patient had an edematous, nonerythematous right fourth proximal interphalangeal (PIP) joint. Urgent irrigation, debridement, and exploration of the right hand into the tendon sheath were performed. With his history of syphillis, an RPR was done, which was reactive with a titer of 1 : 64. A confirmatory FTA-ABS test was completed, rendering a positive result. Based on his history of untreated syphilis, dormancy followed by clinical scenario of swelling of the right fourth finger, and a high RPR titer, he was diagnosed with secondary syphilis manifesting as tenosynovitis.

## 1. Introduction

Tenosynovitis, inflammation of a tendon and its synovial sheath, is a rare manifestation of secondary syphilis that if diagnosed early is entirely reversible. The causes of tenosynovitis can be divided into those of noninfectious or infectious etiology. Examples of noninfectious tenosynovitis include de Quervain's and stenosing tenosynovitis (trigger-finger). Infectious tenosynovitis is often caused by bacterial inoculation of the tendon via direct trauma, contiguous spread, or hematogenous spread.

## 2. Case Presentation

A 52-year-old male with a past medical history of untreated syphilis presented with gradual onset of swelling and pain of the right fourth finger, as well as palmar tenderness proximal to the right fourth metacarpophalangeal joint (MCP). He denied any allergies, surgeries, recent trauma, or fevers. He did report a history of a painless penile lesion after having sexual intercourse with a new partner approximately five months ago. The lesion had resolved spontaneously after being treated with sulfamethoxazole/trimethoprim by his primary care physician. Rapid plasma reagin (RPR) was ordered. A few days later, the result came back positive with a high titer. However, since the patient was asymptomatic, he was lost to follow-up for additional treatment.

**Figure 1:**
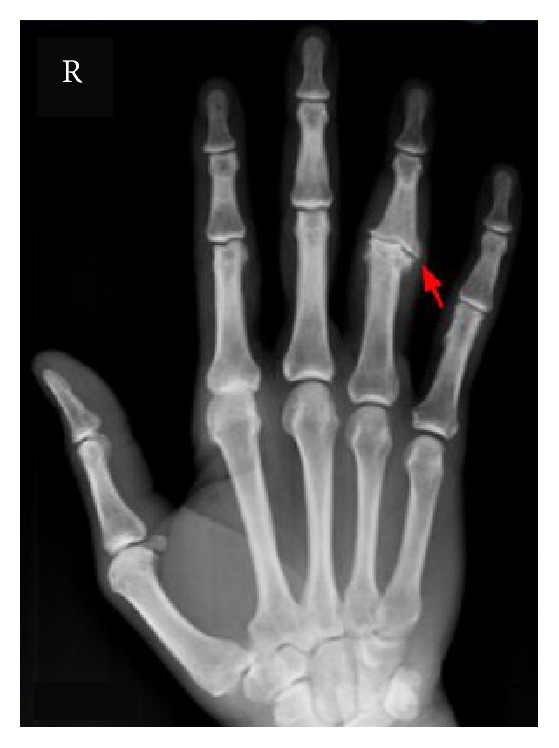
X-ray of right hand, severe degenerative changes at the fourth PIP joint with medial subluxation of the middle phalanx, and periosteal reaction.

At the time of this case presentation, patient had an edematous, nonerythematous right fourth PIP joint. There was tenderness to palpation of the right fourth PIP, extending proximally to the MCP joint. A tender, nonerythematous nodule was appreciated upon palpation of the hand proximal to the right fourth MCP joint. The patient was started on broad spectrum antibiotics to be treated empirically for possible infectious tenosynovitis. X-ray of right hand (Figure 1) showed severe degenerative changes at the fourth proximal interphalangeal joint with medial subluxation of the middle phalanx, subchondral cyst formation, and periosteal reaction. Urgent irrigation, debridement, and exploration of the right hand into the tendon sheath and exploration of all digits of the tendon were performed by plastic surgery.

**Figure 2:**
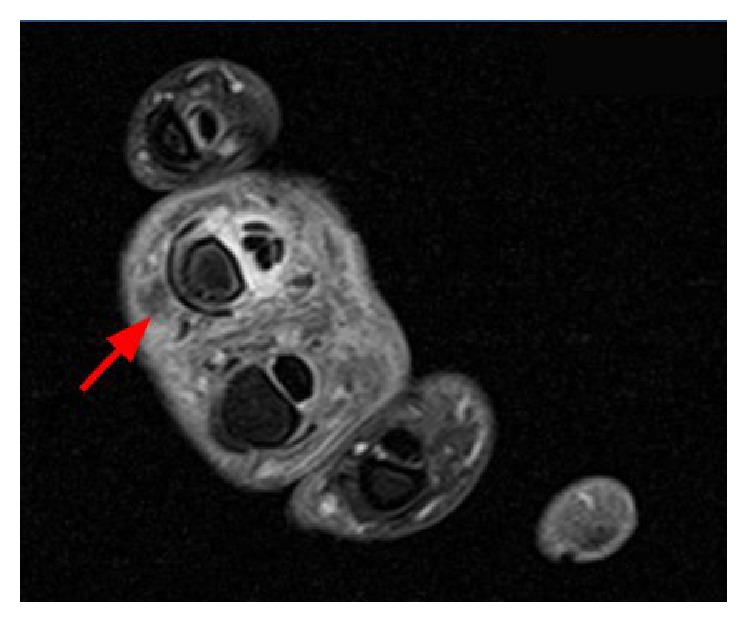
MRI axial view, postcontrast tenosynovitis.

**Figure 3:**
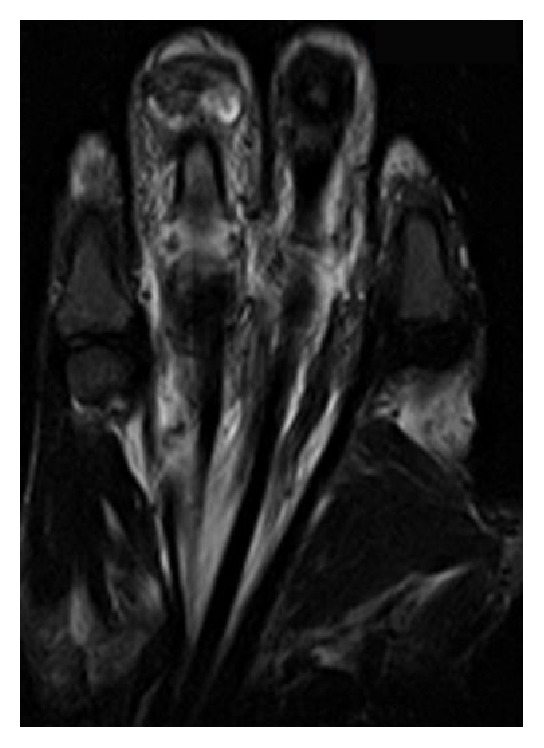
MRI coronal view, edema along tendons.

After surgery, magnetic resonance imaging (MRI) (Figures 2 and 3) of the right hand showed severe degenerative changes of the right fourth PIP with soft tissue edema extending along the palmar aspect of the digit into the wrist and tenosynovitis of the concerned flexor pollicis longus tendon. Blood cultures were negative for bacterial growth, white blood count was 7.8 × 10^9^/L, and patient was afebrile. Due to his history of syphilis infection, Hepatitis, HIV, and RPR were ordered. HIV and hepatitis panels were negative, but RPR was reactive with a titer of 1 : 64. Fluorescent treponemal antibody absorption (FTA-ABS) test was completed, rendering a positive result, and confirming the diagnosis of syphilis.

Based on his history of untreated syphilis followed by a dormancy period, his clinical presentation, and laboratory findings, the patient was diagnosed with secondary syphilis manifesting as tenosynovitis. He was given a dose of benzathine penicillin 2.4 million units IM, and his partner was encouraged to undergo further testing for syphilis.

As an outpatient, he followed up with occupational therapy for whirlpool treatments with good improvement in his range of motion. Complete resolution of symptoms was achieved four weeks after being discharged from the hospital.

## 3. Discussion

While any tendon can be targeted in tenosynovitis, the wrist and hand are most commonly affected. The causes of tenosynovitis can be divided into noninfectious or infectious etiology. Examples of noninfectious tenosynovitis include de Quervain's syndrome and stenosis tenosynovitis (trigger-finger). Infectious tenosynovitis is caused by bacterial inoculation of the tendon via direct trauma, contiguous spread, or hematogenous spread [1].

Infectious tenosynovitis can present in a variety of ways. Pang et al. conducted a retrospective study on 75 patients with tenosynovitis and found that the most common presenting symptom was digit swelling. Other presenting symptoms included pain with passive finger extension, a flexed finger posture, and tenderness to digit palpation [1]. Late signs of tenosynovitis that may present are crepitus and swelling localized to the sheath of the infected tendon.

The diagnosis of tenosynovitis is made based on history and clinical presentation. However, MRI can aid in making the diagnosis and assessing damage of associated joint. With tenosynovitis, an MRI will show thickening of the tenosynovium [2]. Compared to conventional radiography, MRI is more sensitive in the detection of tenosynovium inflammation and bony erosion [3]. Alternatively, ultrasound is a modality that is highly sensitive at detecting tenosynovitis. However, it is not as sensitive as MRI in detecting bony erosions [4].

The literature on syphilis induced tenosynovitis, a form of infectious tenosynovitis, is scarce. A literature review performed using the keywords “tenosynovitis” and “syphilis” returned two studies from 1979 and 1984 [5, 6]. Musculoskeletal complaints, such as tenosynovitis, were observed in up to one-third of patients with secondary syphilis [5]. It involved a variety of joints including the wrists, fingers, knees, and ankles. Patients also presented with arthritis with effusions of the tendon sheaths without erythema or tenderness [5]. On physical exam, most of the patients in these studies had generalized lymphadenopathy and a generalized nonpruritic papulosquamous rash [6]. In both studies, treatment of patients with penicillin G led to rapid resolution of musculoskeletal symptoms [5, 6].

This case is an example of infectious tenosynovitis caused by secondary syphilis. Syphilis is a chronic venereal disease with varied and often subtle clinical manifestations. In 2013, the number of primary and secondary syphilis cases reported to the CDC was 17,375, a 10.9% increase from 2012 [7]. However, the rates of syphilis are likely much higher. Primarily due to variations of completeness and accuracy of reporting, reported rates are generally varied and imprecise. Even in the United States, where the importance of reporting is emphasized and rates have been followed for years, it is estimated that as few as half of the actual cases are reported [8].

The causative agent of syphilis is the bacterium* Treponema pallidum*, hereafter referred to as* T. pallidum*. These spirochetes are typically transmitted via direct contact with an infected lesion, entering the host through disrupted epithelium at sites of minor trauma during sexual intercourse [8]. Untreated, acquired syphilis progresses through three stages, with each stage displaying distinct clinical and pathologic manifestations. Primary syphilis, the earliest stage, manifests approximately three weeks after exposure. It is defined by the presence of a firm, nontender, raised, red lesion, known as a chancre, at the site of invasion on the penis, cervix, vaginal wall, or anus. This characteristic chancre heals within three to six weeks with or without therapy [4].

Hematogenous dissemination of* T. pallidum* causes the widespread findings in secondary syphilis, characterized by mucocutaneous and multisystem involvement. The skin lesions of this stage are maculopapular, scaly, or pustular in nature, typically appearing on the palms or soles of the feet. Depending on the type of surface, lesions may assume various appearances. Moist areas of the skin, such as the anogenital region, inner thighs, and axillae may show condylomata lata, broad-based, elevated plaques. Silvery-gray superficial erosions form on mucous membranes, particularly those in the mouth, pharynx, and external genitalia. As in primary syphilis, the lesions of secondary syphilis are superficial and painless and contain the inciting spirochetes. They too are infectious. Lymphadenopathy, mild fever, malaise, and weight loss are also common to this stage. Symptoms of secondary syphilis may last several weeks. Even without treatment, the signs of primary and secondary syphilis resolve spontaneously, and patients then enter the latent stage of infection [4].

After a variable period of latency, usually five years or more, the manifestations of the tertiary stage develop in approximately one-third of untreated patients [4]. There are three main manifestations, which may occur alone or in combination: cardiovascular syphilis, neurosyphilis, and benign tertiary (gummatous) syphilis. Syphilitic aortitis, a form of cardiovascular syphilis, accounts for the majority of cases of tertiary syphilis. Symptomatic neurosyphilis manifests in a variety of ways, including chronic meningovascular disease, tabes dorsalis, and a general paresis. Benign tertiary syphilis is characterized by the formation of gummas, white-gray rubbery lesions, in various sites throughout the body. Although any organ can be affected, gummas occur primarily in skin, mucous membranes, subcutaneous tissue, bone, and joints. Since the use of effective antibiotics, gummas are now very rare and are seen mainly in individuals with acquired immune deficiency syndrome (AIDS) [4].

While clinical staging is useful in guiding therapeutic decisions, it is imprecise. Patients with late stages of disease may have no recollection of signs of earlier stages, possibly because most syphilitic lesions are painless or because some patients may not have clinically apparent primary or secondary lesions. Of note, there is considerable overlap between stages [8].

*T. pallidum* is too slender to be detected using Gram stain, although it can be visualized by silver stain, dark-field examination, and immunofluorescence. Therefore, serologic testing is the most widely used laboratory technique for the diagnosis of syphilis [8, 9]. Serologic testing should include the use of both nontreponemal and treponemal tests. Either test can be used as the initial screening test. Confirmatory testing is necessary due to the potential for a false positive screening test result. For example, false positive VDRL tests results are not uncommon and are often seen in association with acute infections, collagen vascular diseases, drug addiction, pregnancy, hypergammaglobulinemia, and lepromatous leprosy [4]. Nontreponemal tests, which test for reagin antibodies, are based upon the reactivity of serum from infected patients to a cardiolipin-cholesterol-lecithin antigen. Although the nontreponemal tests are nonspecific, they have traditionally been used for initial syphilis screening due to their relatively low cost, ease of use, and ability to be quantified for the purpose of following response to therapy. Such tests include Rapid Plasmin Reagin (RRR), Venereal Disease Research Laboratory (VDRL), and Toluidine Red Unheated Serum Test (TRUST). Treponemal tests have historically been more complex and expensive to perform than nontreponemal tests. Therefore, they have traditionally been used as confirmatory tests for syphilis when the nontreponemal tests are reactive. However, newer automated versions of these tests enhance simplicity and facilitate ease of use. As a result, these tests are increasingly used to screen for syphilis, rather than as confirmatory tests. Such tests include the Fluorescent treponemal antibody absorption (FTA-ABS), Microhemagglutination test for antibodies to* T. pallidum* (MHA-TP), and* T. pallidum* enzyme immunoassay (TP-EIA). As a group, these tests are based upon the detection of antibodies directed against specific treponemal antigens; therefore, they tend to be more specific than nontreponemal tests. The TP-EIA test has become the favored treponemal test in many laboratories, particularly those with large volumes of testing. Although both nontreponemal and treponemal tests are subject to occasional false positive results in patients without syphilis, sequential use of the two tests greatly improves the accuracy of serologic diagnosis [8].

The treatment of choice for* T. pallidum* is penicillin G with a goal of achieving and maintaining serum penicillin concentration of more than 0.03 ug per mL for 7–14 days. Typically, this can be achieved by a single IM injection of 2.4 million units of benzathine penicillin G in patients with primary, secondary, and early latent syphilis. For patients with penicillin allergies or lacking access to IM penicillin, a single, 2-gram oral dose of azithromycin has been shown to be equally effective in treating primary and secondary syphilis [10].
